# Quantitative Rapid Magnetic Immunoassay for Sensitive Toxin Detection in Food: Non-Covalent Functionalization of Nanolabels vs. Covalent Immobilization

**DOI:** 10.3390/toxins16010005

**Published:** 2023-12-20

**Authors:** Alexey V. Orlov, Sergey L. Znoyko, Juri A. Malkerov, Artemiy M. Skirda, Denis O. Novichikhin, Alexandra S. Rakitina, Zoia G. Zaitseva, Petr I. Nikitin

**Affiliations:** 1 Prokhorov General Physics Institute of the Russian Academy of Sciences, 38 Vavilov Street, 119991 Moscow, Russia; znoykos@yandex.ru (S.L.Z.); jurimalkerov@yandex.ru (J.A.M.); artemskirda@mail.ru (A.M.S.); nammen@yandex.ru (D.O.N.); sasha080700@gmail.com (A.S.R.); zajzoya@yandex.ru (Z.G.Z.); 2 National Research Nuclear University MEPhI (Moscow Engineering Physics Institute), 31 Kashirskoe Shosse, 115409 Moscow, Russia; 3 Moscow Institute of Physics and Technology, 1A Kerchenskaya Street, 117303 Moscow, Russia

**Keywords:** food safety, toxin detection, magnetic lateral flow immunoassay, non-covalent magnetic labels, covalent immobilization, magnetic particle quantification, antibody sorption density, zearalenone, Fusarium graminearum, analytical performance

## Abstract

In this study, we present a novel and ultrasensitive magnetic lateral flow immunoassay (LFIA) tailored for the precise detection of zearalenone, a mycotoxin with significant implications for human and animal health. A versatile and straightforward method for creating non-covalent magnetic labels is proposed and comprehensively compared with a covalent immobilization strategy. We employ the magnetic particle quantification (MPQ) technique for precise detection of the labels and characterization of their functionality, including measuring the antibody sorption density on the particle surface. Through kinetic studies using the label-free spectral phase interferometry, the rate and equilibrium constants for the binding of monoclonal antibodies with free (not bound with carrier protein) zearalenone were determined to be k_on_ = 3.42 × 10^5^ M^−1^s^−1^, k_off_ = 7.05 × 10^−4^ s^−1^, and K_D_ = 2.06 × 10^−9^ M. The proposed MPQ-LFIA method exhibits detection limits of 2.3 pg/mL and 7.6 pg/mL when employing magnetic labels based on covalent immobilization and non-covalent sorption, with dynamic ranges of 5.5 and 5 orders, correspondingly. We have successfully demonstrated the effective determination of zearalenone in barley flour samples contaminated with *Fusarium graminearum*. The ease of use and effectiveness of developed test systems further enhances their value as practical tools for addressing mycotoxin contamination challenges.

## 1. Introduction

Mycotoxins, a class of toxic secondary metabolites produced by various molds, pose a significant threat to public health due to their ubiquity and potential for contamination in food and feed supplies [1,2,3]. Among these mycotoxins, zearalenone stands out as one of the most prevalent and perilous compounds [4,5]. Zearalenone, a mycotoxin produced by Fusarium species, is commonly found in cereals such as maize, barley, oats, wheat, and rice [6]. This toxin’s prevalence is notable, particularly in humid and temperate climates that favor Fusarium growth [7]. Chemically, zearalenone is a nonsteroidal estrogenic mycotoxin, structurally similar to estradiol, and is known for its xenoestrogenic effects, potentially disrupting endocrine functions in both animals and humans [8,9]. Chronic exposure poses significant risks, especially in livestock, leading to reproductive disorders and immunological issues [4]. The LD50 of zearalenone varies among species; for rodents, it ranges from several hundred milligrams to over a gram per kilogram of body weight, underscoring its low acute toxicity [10]. However, the main concern with zearalenone lies in its chronic endocrine-disrupting effects rather than in its immediate toxicity. Its precise quantitative determination is of paramount importance in ensuring food safety and safeguarding public health [11,12]. In many countries, zearalenone content in food products is meticulously regulated by legislation [13,14]. The specific values of maximum residue limits (MRLs) vary by country, and the most stringent limits, such as the EU regulation’s MRL of 20 ng/g [14], are typically applied to food products intended for children and infants.

In the pursuit of accurate zearalenone detection, a multitude of methods have been developed, including high-performance liquid chromatography (HPLC), gas chromatography-mass spectrometry (GC-MS), and enzyme-linked immunosorbent assays (ELISA) [15,16,17,18,19]. These methods, while effective, have their limitations and are generally not suitable for rapid, on-site testing.

Within the realm of zearalenone detection, lateral flow immunoassay (LFIA) is being considered as a promising approach [20,21,22]. LFIA offers simplicity, portability, and cost-effectiveness, making it well-suited for on-site applications [23,24,25]. In particular, a diverse range of LFIA-based approaches has been developed for zearalenone detection, employing various labels and utilizing different optical methods, including colorimetric assays (e.g., in combination with gold nanoparticles) [26,27,28], fluorescent assays (e.g., using quantum dots or other fluorescent reporters) [29,30,31,32,33,34], surface-enhanced Raman spectroscopy [35,36], and others [37,38,39,40]. However, the challenge of developing LFIA-based methods for the accurate quantitative determination of zearalenone in real samples under field conditions, while maintaining analytical characteristics comparable to traditional laboratory methods, remains unresolved. Notably, electronic (non-optical) detection methods, specifically those utilizing magnetic nanolabels in combination with the magnetic particle quantification (MPQ) technique [41,42,43], hold significant potential for enhancing sensitivity and accuracy of LFIA.

One of the key challenges in developing effective, quantitative, and reproducible LFIA is nanolabel functionalization. Typically, this involves creating conjugates in which nanoparticles are bound with specific antibodies. Existing methods for particle functionalization involve chemical conjugation, which includes the use of crosslinkers based on carbodiimide, hydroxysuccinimide, maleimide, and other compounds, as well as physical sorption techniques [44,45,46,47,48]. However, universally applicable and easy-to-use methods for creating reproducible functionalized nanolabels for the highly sensitive detection of low-molecular-weight compounds, such as zearalenone, along with techniques for the comprehensive functional characterization of these conjugates, are still to be developed.

This study aims to address the mentioned challenges and introduces a highly sensitive magnetic LFIA for precise detection of zearalenone using a versatile and straightforward method for creation of non-covalent magnetic labels compared with those obtained with a covalent immobilization strategy. Using MPQ, we conducted a comprehensive functional characterization of the magnetic labels, including the quantification of antibody sorption density on conjugate surfaces. Notably, the development of the magnetic LFIA was preceded by in-depth kinetic studies of immunoreagents, which included determining the binding constants of monoclonal antibodies to free zearalenone (not conjugated with a carrier protein). We optimized the key parameters of the LFIA test system, assessed its analytical characteristics, demonstrated the advantages of the proposed approach over existing alternatives, and demonstrated its capability for detecting zearalenone in real samples.

## 2. Results and Discussion

### 2.1. Characterization of Monoclonal Antibodies against Zearalenone

The initial series of experiments was conducted to investigate a crucial parameter influencing the analytical properties of immunosensors—namely, performed the kinetic characterization of monoclonal antibodies that recognize zearalenone (anti-ZEA Ab). For the purpose, their equilibrium and kinetic constants for association and dissociation with the target analyte were determined using the label-free methods of spectral-phase interferometry and spectral-correlation interferometry (see Section 4.2 of the Materials and Methods) [49,50,51]. Sensor chips were fabricated with conjugates of the carrier protein (BSA) and zearalenone immobilized on their surfaces.

**Figure 1:**
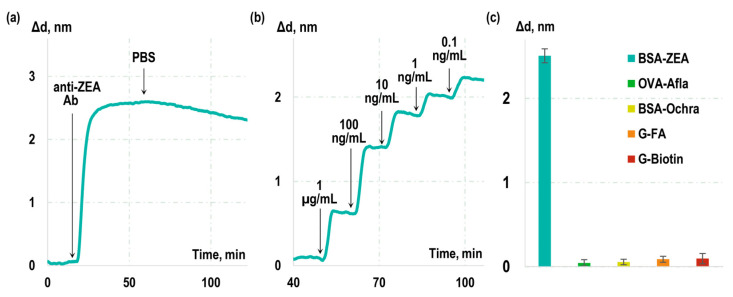
Kinetic characterization and assessment of specificity of monoclonal antibodies to zearalenone: (**a**) real-time sensorgram demonstrating the interaction between anti-ZEA antibodies and the BSA-ZEA conjugate; (**b**) interaction of anti-ZEA antibodies with BSA-ZEA conjugates, and (**c**) with non-target molecules: ochratoxin A (Ochra), aflatoxin B1 conjugated with ovalbumin (OVA-Alfa), biotin, and folic acid conjugated with gelatin (G-Biotin and G-FA).

A solution of monoclonal antibodies to ZEA was passed over the chip surface, and, during the first stage, the real-time changes in the biolayer thickness (sensorgram) due to formation of ‘BSA-ZEA–anti-ZEA Ab’ immunocomplexes were recorded (see Figure 1a). Subsequently, a solution without anti-ZEA Ab was added, and a sensorgram representing dissociation of the formed immunocomplexes was recorded. Our findings revealed that the rate constants for the association (k_on_) and dissociation (k_off_) for this interaction were 2.18 × 10^4^ M^−1^s^−1^ and 5.08 × 10^−4^ s^−1^, respectively. The equilibrium dissociation constant (K_D_) was determined to be 23.3 nM.

Notably, the obtained constants describe the interaction of anti-ZEA antibodies with BSA-ZEA rather than with free ZEA in solution. To assess the kinetic characteristics of antibody interaction specifically with free ZEA, label-free real-time sensorgrams were recorded during competitive interactions (Figure 1b). Known concentrations of ZEA were introduced into the antibody solution, and the interaction between anti-ZEA antibodies and BSA-ZEA was monitored in real time. Subsequently, the kinetic characteristics of anti-ZEA antibody interaction with free ZEA were calculated based on the effect of free ZEA on the previously measured interaction constants with BSA-ZEA. The resulting kinetic and equilibrium constants for this interaction were determined to be as follows: k_on_ = 3.42 × 10^5^ M^−1^s^−1^, k_off_ = 7.05 × 10^−4^ s^−1^, and K_D_ = 2.06 nM.

Table 1 presents a comparison of the measured constants that describe the interactions between antibodies and BSA-ZEA, as well as free ZEA. As it is evident from the table, interactions with free ZEA exhibit significantly more favorable characteristics, namely, a higher kinetic association constant and a lower kinetic dissociation constant. This phenomenon can be explained by the fact that, in the case of the free ZEA target, these constants describe the interaction of antibodies with the free small molecules in solution. In contrast, in the case of the BSA-ZEA target, the interaction occurs with zearalenone integrated within the BSA-ZEA conjugate immobilized on the surface of the sensor chip. Several factors could contribute to these distinctions, including potential steric shielding of zearalenone within the conjugate, and reduced potential for reorientation during the interaction process.

Furthermore, the specificity of the used monoclonal antibodies was assessed. For this purpose, sensor chips were fabricated with immobilized BSA conjugates with non-target molecules and mycotoxins, specifically ochratoxin A, aflatoxin B1, biotin, and folic acid. Then, a solution of anti-ZEA antibodies was passed over the chip surface, and the related increase in the sensorgram was recorded. The obtained experimental curves, along with the calculated values of Δd indicating the growth of the biolayer on the glass surface, are presented in Figure 1c. Our observations reveal a substantial increase of 2.5 ± 0.2 nm in the sensorgram when antibodies interacted with the BSA-ZEA conjugate. Importantly, the growth in the sensorgram due to their cross-reactivity with the examined non-target toxins and molecules remained negligible.

### 2.2. Synthesis and Characterization of Magnetic Labels for LFIA

**Figure 2:**
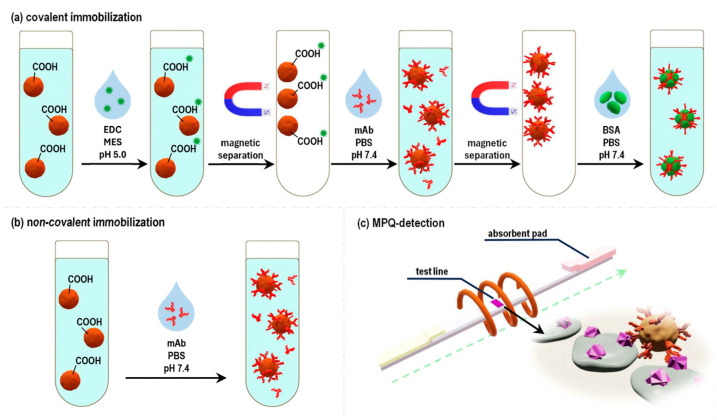
Scheme for obtaining functionalized magnetic LFIA labels using covalent (**a**) and non-covalent (**b**) immobilization of anti-ZEA Ab; and a scheme for investigation of functional characteristics of the obtained magnetic conjugates using quantitative magnetic immunochromatography (**c**).

The next series of experiments was dedicated to the creation of functionalized LFIA labels, specifically, the “magnetic particle-anti-ZEA Ab” labels. For that purpose, commercially available carboxylated (-COOH) polystyrene magnetic particles (MPs) with a diameter of 200 nm were utilized (see Section 4.1 of the Materials and Methods). These particles were functionalized with anti-ZEA antibodies using two different methods: (i) covalent immobilization by the standard carbodiimide method (Figure 2a) and (ii) non-covalent adsorption of immunoglobulins onto the particle surface (Figure 2b).

Initially, the particles were characterized with SEM and DLS (Figures S1 and S2). Then, to analyze the quantity of antibodies immobilized as a result of the functionalization process on the surface of particles, the bicinchoninic acid (BCA) protein assay was used. The quantitative control of the protein concentration was carried out in the following samples: the antibody solution added to MPs before immobilization and the antibody solution remaining after completion of the immobilization process and the removal of the MPs via magnetic separation. The difference in these concentrations was used to calculate the amount of antibodies remaining on MPs as a result of immobilization. To accurately assess the amount of antibodies non-covalently immobilized on MPs in these experiments, the unbound antibodies were magnetically washed from samples. Furthermore, the LFIA labels obtained through covalent conjugation were not blocked with BSA solution, as it was for other experiments. Using this technique, it was determined that the use of non-covalent immobilization resulted in magnetic labels with an antibody sorption density of 2.2 µg of anti-ZEA Ab per 1 mg MP, while the labels obtained by the covalent conjugation had 3.1 µg of anti-ZEA Ab per 1 mg MP.

Thus, the non-covalent sorption of antibodies is not significantly (only by 29%) inferior to the covalently immobilized antibodies. It is worth noting that the used anti-ZEA antibodies were of the IgG1 subclass and were characterized by a relatively high isoelectric point (pI). According to the literature, the pI for IgG1 is approximately 8.6 [52]. Consequently, at neutral pH values, immunoglobulin molecules carry a positive charge, which enhances the efficiency of non-covalent sorption on magnetic particles modified with negatively charged carboxyl groups. To prevent potential dissociation of non-covalent nanobioconjugates, they were stored in a solution containing anti-ZEA Ab antibodies, along with a preservative (sodium azide). This ensured a dynamic equilibrium between the unbound and surface-immobilized antibody molecules.

**Figure 3:**
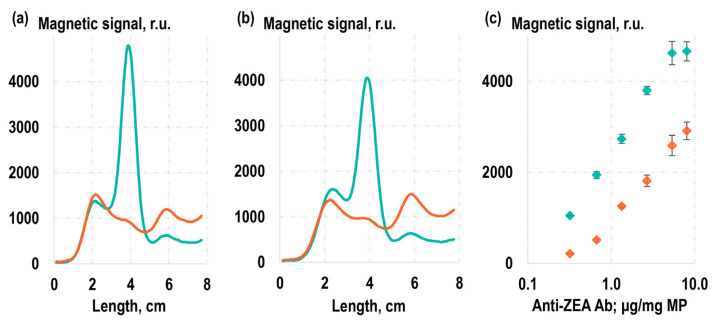
Experimentally registered distribution of magnetic conjugates along an LFIA strip, recorded using the MPQ method for magnetic conjugates obtained by covalent (**a**) and non-covalent (**b**) immobilization of antibodies while detecting samples in the absence of ZEA (cyan line) and in the presence of ZEA at 100 ng/mL (orange line) in the analyzed sample; and determination of the amount of active antibodies via magnetic LFIA in two dilution series, one of which maintains a constant total concentration of target (cyan dots) and non-target (orange dots) antibodies during non-covalent immobilization (**c**).

The next series of experiments involved an examination of the functional characteristics of the obtained magnetic labels, specifically their capacity to perform their recognition function and bind with the target antigen, ZEA. To achieve this, quantitative magnetic LFIA was employed (Figure 2c). In that process, a LFIA strip was placed into an analyzed solution containing the magnetic labels. The BSA-ZEA conjugate was immobilized on the strip to form a test line (TL). As the solution migrated along the strip under the capillary forces, a portion of the magnetic labels that was captured by the target formed a TL, hosting bound magnetic labels. The magnetic labels that were not bound to the target and, therefore, were not captured by the test line proceeded to the absorbent pad (AP). Subsequently, the distribution of magnetic labels along the LFIA strip was quantitatively recorded using the highly sensitive MPQ method. The results of these experiments showed that 61% of the magnetic labels obtained by the covalent immobilization of antibodies were retained at the TL, with only 16% reaching the AP (Figure 3a). For the magnetic conjugates obtained via non-covalent sorption of antibodies, these values were 57% for TL and 17% for AP (Figure 3b). These values suggest that the majority of labels carry functionally active antibodies, ensuring effective binding to the TL. Notably, since the conjugates obtained using the covalent immobilization followed standard protocols recommended by the manufacturer, we focused our particular interest on the more comprehensive examination of the functional characteristics of the conjugates acquired through the method of non-specific sorption.

For a more detailed functional characterization of labels obtained through the method of non-specific sorption of antibodies, two sets of labels were prepared. The first set was obtained by adding anti-ZEA Ab to the MPs at various concentrations (Figure 3c). The second set was similar to the first, but in addition to anti-ZEA Ab, non-target antibodies were also introduced to the MPs in a manner that maintained a constant total antibody concentration during the sorption process. Following the preparation, the labels were purified to remove unbound antibodies using magnetic washing. Subsequently, they underwent magnetic LFIA, and the magnetic signal on TL, composed of BSA-ZEA, was recorded. With these two sets of labels, two calibration curves were plotted as the relationship between the magnetic signal on TL and the concentration of anti-ZEA Ab added during the immobilization process. Then, using the model described in [53], the amount of active antibodies achieved during such non-covalent sorption was estimated based on the correlation between these curves. The value obtained was 53 ng of antibodies per 1 cm^2^ of particle surface, or, given the 200 nm particle size, approximately 270 active antibodies per particle. These values agree with the characteristic values achieved during the sorption of class G immunoglobulins on polystyrene surfaces [54,55]. In addition, the presence of multiple antibodies on a magnetic nanoparticle determines its polyvalency, and the values of the kinetic association constants of such conjugates are several orders of magnitude higher than those of molecular antibody association with antigen.

### 2.3. Optimization of Magnetic Lateral Flow Immunoassay Parameters

**Figure 4:**
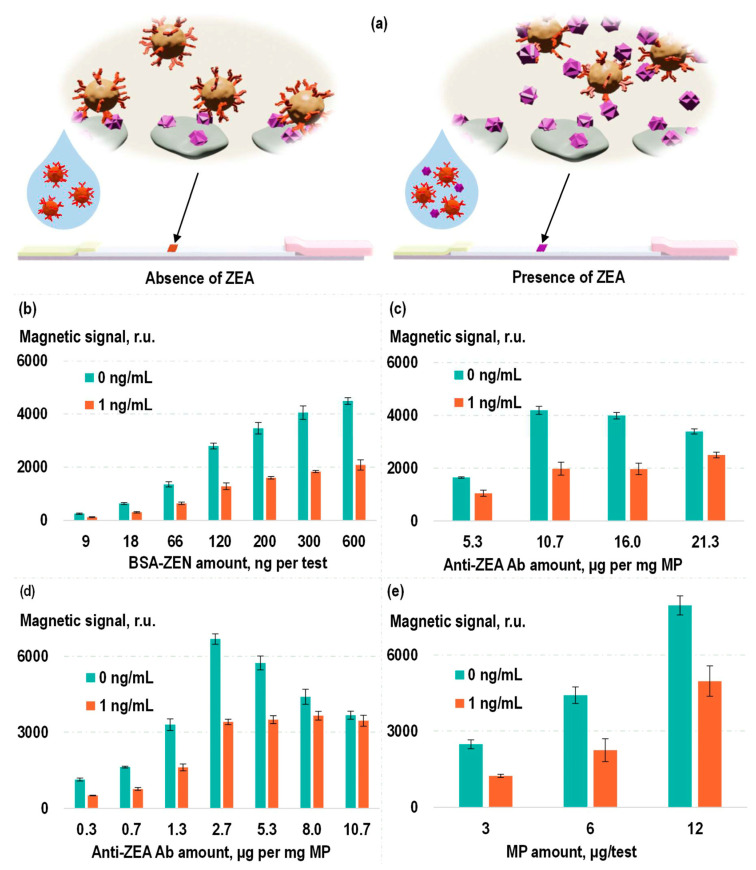
Scheme of the competitive magnetic lateral flow immunoassay (**a**) and experimental optimization of its parameters: quantity of BSA-ZEN on TL (**b**); quantity of anti-ZEA Ab used in label preparation via covalent (**c**) and non-covalent (**d**) antibody immobilization; quantity of magnetic labels in a single test (**e**).

To establish a sensitive and specific test system for the detection of zearalenone, a competitive format of magnetic LFIA was used. Magnetic labels functionalized with anti-ZEA Ab were introduced into the analyzed samples containing ZEA. Then, this solution was applied to an LFIA strip containing BSA-ZEA on the TL. As demonstrated earlier, in the absence of ZEA in the analyzed sample, the magnetic labels, composed of MP–anti-ZEA Ab, specifically bind to BSA-ZEA on the TL, resulting in the magnetic signal in the TL area of the strip. The presence of ZEA in the analyzed sample due to its binding to antigen-recognition antibody fragments on the magnetic labels diminishes the probability of the label binding to the TL. That leads to a reduction in the detected magnetic signal on the TL (Figure 4a). The following parameters were optimized: quantity of magnetic labels, quantity of anti-ZEA Ab per TL, and quantity of BSA-ZEN per TL (see Figure 4b–e). This optimization process aimed at a balance that provided both high efficiency in the conjugate binding to the TL and a sensitive ZEA-dependent reduction in the efficiency of this binding.

In the optimization process, the criterion was the ratio of magnetic signal in the TL region when analyzing samples without ZEA to the magnetic signal under analysis of samples containing ZEA at a relatively low concentration (1 ng/mL). As can be seen in Figure 4b, an increase in the BSA-ZEN quantity on the TL results in an elevation of the magnetic signal on the TL without diminishing the mentioned ratio. Consequently, for subsequent experiments, the highest of the examined concentrations of BSA-ZEN was employed (0.6 µg of BSA-ZEN per test).

In Figure 4c, the results of optimizing the quantity of anti-ZEA Ab used in the production of labels via covalent immobilization are presented. It is evident from the figure that the magnetic signal in the TL region also increases with the rising quantity of antibodies. However, the ratio of magnetic signals obtained during the analysis of ZEA at concentrations of 0 and 1 ng/mL exhibits a bell-shaped dependence. This can be explained by the fact that, on the one hand, when there is an insufficient quantity of antibodies, the magnetic signals are relatively low. On the other hand, in the case of an excess of antibodies per magnetic particle, a smaller portion of such labels will bind to free ZEA in a solution with a low analyte concentration, which leads to a decrease in the sensitivity of the competitive assay. Therefore, for subsequent experiments, the optimal ratio among those examined was employed: 10.7 µg of anti-ZEA Ab per 1 mg MP. Similarly, the quantity of magnetic labels used in a single test also had an optimal value, which was equivalent to 6 µg of “MP–anti-ZEA Ab” labels per test (Figure 4e).

The dependences examined during selection of the quantity of anti-ZEA Ab used for production of labels via non-covalent sorption are even more intriguing (Figure 4d). Since, in this case, there was no washing process to remove unbound antibodies from the particles, the dependence of magnetic signal in the TL region on antibody quantity was not monotonic. In the low concentration range (0.3–2.7 µg anti-ZEA Ab per 1 mg MP), an increase in antibody concentration results in a growth of magnetic signals. Presumably, at such antibody concentrations, they are in deficit and largely adsorb onto the particles. The remaining unbound antibodies have a limited impact on the results of magnetic LFIA detection. However, in the high concentration range (2.7–10.7 µg of anti-ZEA Ab per 1 mg MP), a decline in magnetic signals is observed even when analyzing samples that do not contain ZEA. This could be attributed to the fact that at higher concentrations, the quantity of antibodies immobilized on the particles has already reached saturation, and further increases are marginal. Consequently, an increasing concentration of antibodies becomes present in the form of unbound molecules, which can specifically interact with the TL, effectively reducing the likelihood of conjugate binding to it. Nevertheless, the optimal concentration can simultaneously ensure a relatively high antibody sorption density and, on the other hand, a small proportion of unbound molecules. In our experiments, the concentration selected for the further experiments according to the previously chosen criterion was 2.7 µg anti-ZEA Ab per 1 mg MPs.

### 2.4. Study of the Analytical Characteristics of the Developed Magnetic LFIA Systems

**Figure 5:**
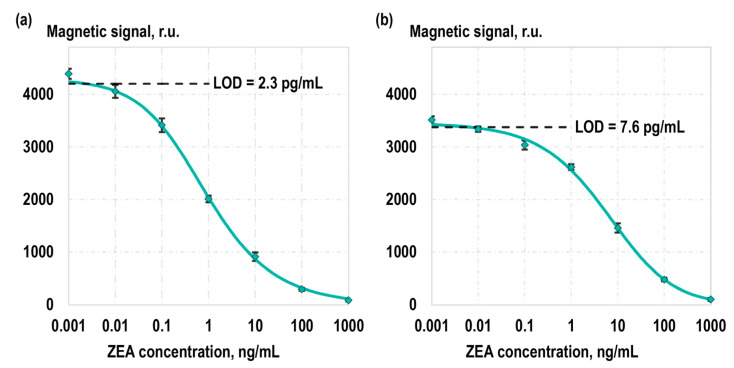
Calibration curves as dependences of the magnetic signal at the TL on ZEA concentration in calibration samples using magnetic labels obtained by covalent (**a**) and non-covalent (**b**) antibody immobilization.

Following the optimization of experimental parameters, calibration curves representing the dependence of the magnetic signal detected on the TL on the concentration of ZEA in calibration samples were obtained (Figure 5). Separate calibration curves were obtained using magnetic labels with covalent antibody immobilization and non-covalent sorption. In both cases, calibration samples with the following ZEA concentrations were utilized: 1 µg/mL, 100 ng/mL, 10 ng/mL, 1 ng/mL, 100 pg/mL, 10 pg/mL, 1 pg/mL, and 0 pg/mL. The concentration of ZEA in the calibration samples was pre-validated with the liquid chromatography–tandem mass spectrometry. The calibration curves were fitted using a five-parameter logistic curve. The limits of detection (LOD), determined based on the 2σ criterion, were 2.3 pg/mL and 7.6 pg/mL when using the covalent and non-covalent magnetic labels, respectively. The limits of quantification (LOQ), determined using a 10σ criterion, were 21 pg/mL and 62 pg/mL, respectively. The dynamic ranges were 5.5 and 5 orders of magnitude, and the linear detection ranges (on a log-log scale) covered 3 and 4 orders of magnitude for covalent and non-covalent labels, respectively. The assay time in both cases was 25 min including the registration of the magnetic signal using an MPQ reader for the magnetic particles. We particularly highlight the high reproducibility of our method: the average relative standard deviation (RSD) of the detected signals in the low concentration range (less than 1 ng/mL) is 3%, and, in the high concentration range (1–100 ng/mL), the RSD is 7%.

We hypothesize that a key factor contributing to the different detection performances is our approach of not washing unbound antibodies in the non-covalent sorption method. This results in a slight decrease in sensitivity, yet the performance remains superior to existing methods. Importantly, this approach provides a crucial advantage: the conjugates in dynamic equilibrium are minimally affected by desorption processes, which could be critical in the case of non-covalent antibodies binding to particles. Another factor that may contribute to the distinct characteristics of covalent and non-covalent conjugates is the previously calculated difference in antibody sorption density: 2.2 µg and 3.1 anti-ZEA Ab per 1 mg MP, respectively.

Notably, the developed MPQ-LFIA concept provides advanced capabilities for quality control beyond traditional optical methods. Typically, a control line (CL) is added to verify the successful migration of particles; particles not bound to the test line are captured at the CL. In our case, since the MPQ method allows for the quantitative registration of the particle distribution along the entire volume of the LFIA strip, the quantification of unbound particles is possible without the need for a separate CL. Nevertheless, implementing a CL (e.g., using anti-species-specific secondary antibodies) is feasible, allowing for quantitative analysis of magnetic particles binding to it.

Table 2 presents a comparison of the achieved analytical characteristics of the developed assay systems with alternative LFIA-based approaches. As it is evident from the table, the assay system created using covalent magnetic conjugates excels in analytical characteristics compared to all alternative lateral flow techniques. Additionally, the table shows that the assay system based on the covalent magnetic labels unsurprisingly outperforms those employing the non-covalent ones in terms of analytical characteristics. Nonetheless, the non-covalent approach offers undeniable advantages, primarily in its utmost simplicity for obtaining the non-covalent magnetic labels: their preparation merely necessitates the addition of an antibody solution to the particles. Subsequently, non-covalent labels are ready for long-term storage and/or immediate application, requiring no purification from unbound antibodies. Furthermore, as observed in Table 2, the analytical characteristics achieved with non-covalent conjugates appear highly attractive in comparison to other LFIA-based approaches, especially in terms of the combination of characteristics, including the dynamic range. 

In comparing our proposed method with alternative LFIA approaches (based on colloidal gold, quantum dots, aggregation-induced emission (AIE), etc.), it is noteworthy that our method exhibits superior sensitivity and dynamic range, which can be attributed to the ultra-sensitive electronic registration of magnetic labels using the MPQ technique. Unlike colloidal gold LFIAs, it allows for precise quantitative analysis and offers a broader dynamic range than quantum dot-based assays. Additionally, our method maintains robustness under various environmental conditions, a challenge often encountered by AIE-LFIAs. A potential limitation of our method at present is the need for an MPQ detector to obtain accurate quantitative results. We are confident that scaling up its production and expanding its popularity will alleviate this limitation shortly.

### 2.5. Analysis of Real Samples

To assess the applicability of the developed method for real sample analysis, barley flour samples, both uninfected and infected with *Fusarium graminearum*, were utilized. The zearalenone content in the samples was quantified in an independent laboratory using liquid chromatography–tandem mass spectrometry. The uncontaminated and contaminated flour samples were mixed in various proportions, subjected to zearalenone extraction and sample preparation procedures (see Section 4.7). Then, these samples were analyzed using the developed assay system. The results of this analysis are presented in Table 3, demonstrating a high degree of recovery for both covalent and non-covalent conjugates. This highlights the potential of the developed magnetic assay systems for real sample analysis. A comprehensive validation of the system using a wide range of real contaminated and characterized samples, including corn, wheat, sorghum, and rice, is the objective of our future research.

It should be noted that, in our method, the colored fragments of experimental samples or their extracts do not contribute to the recorded signal, as we utilize a fully electronic detection of labels based on their magnetic properties. In this regard, sample pigmentation will not affect actual sample testing. That is another advantage over traditional optical approaches, where the coloration and autofluorescence of samples may cause issues (e.g., the pigmentation of corn extract could lead to false positive results under optical detection).

## 3. Conclusions

In this study, we have developed a highly sensitive magnetic lateral flow immunoassay for accurate detection of zearalenone, a mycotoxin that poses a significant threat to human and animal health. The assay leverages the use of magnetic labels functionalized with anti-ZEA antibodies, offering the advantage of rapid and accurate quantitative detection. We compared the performance of covalent antibody immobilization with non-covalent sorption methods and comprehensively characterized their functionality. Our kinetic studies revealed essential interaction parameters of anti-ZEA antibodies with free zearalenone, shedding light on the binding constants and equilibrium dissociation constant of interaction with mycotoxin in non-bound with carrier protein form. The proposed MPQ-LFIA method exhibited impressive detection limits, with 2.3 pg/mL and 7.6 pg/mL for covalent and non-covalent magnetic labels, respectively. The dynamic ranges of the two methods encompassed five orders of magnitude, showcasing their versatility in detecting a wide range of zearalenone concentrations. We explored the practicality of our method by analyzing real samples, including barley flour samples infected with *Fusarium graminearum*. The results exhibited a high degree of recovery for both covalent and non-covalent conjugates, showcasing the potential of our developed magnetic LFIA systems for real sample analysis. 

The novelty of our magnetic LFIA lies in its use of non-covalent magnetic labels, which sets it apart from traditional LFIAs that predominantly employ colloidal gold or fluorescent labels. Our approach enables ultra-sensitive detection of labels due to using an MPQ technique for precise quantification of labels from the entire volume of LFIA test strips, which is a significant advance over the capabilities of most existing optical LFIAs. That not only enhances sensitivity but also provides a wider dynamic range, crucial for detecting low-concentration analytes. While retaining the simplicity and rapidity of traditional LFIAs, our method introduces a new level of precision and reliability, making it especially suitable for complex sample matrices where conventional LFIAs might struggle. The developed MPQ-LFIA method is adaptable for detecting various mycotoxins and chemical compounds, contingent on the availability of specific antibodies for the target analytes. By altering the binding agents, like antibodies, this method can be extended to a wide range of substances, offering its advantages of high sensitivity and specificity in diverse applications.

In summary, our findings indicate that the magnetic LFIA systems developed in this study offer a promising solution for the sensitive and specific detection of ZEA in various applications, especially in the food safety industry. The versatility and efficiency of these test systems, combined with their ease of use, make them valuable tools for addressing mycotoxin contamination challenges.

## 4. Materials and Methods

### 4.1. Materials

The reagents utilized in this study included monoclonal antibodies against ZEA (anti-ZEA), conjugates of BSA with zearalenone, ovalbumin with aflatoxin B1, and BSA with ochratoxin A, as well as barley flour samples infected with *Fusarium graminearum* and positive calibrators (DTS Biotech Ltd., Pushchino, Russia). The following chemicals were employed for the modification of microscope cover glasses: 95% ethanol (Ferein, Moscow, Russia), DMSO (Chimmed, Moscow, Russia), APTES (98%, Sigma Aldrich, St. Louis, MO, USA), succinic anhydride (Sigma Aldrich, St. Louis, MO, USA), EDC-HCl (Sigma Aldrich, St. Louis, MO, USA), MES buffer (Sigma Aldrich, St. Louis, MO, USA), and phosphate-buffered saline (PBS) with a pH of 7.4 (PanEco Ltd., Moscow, Russia). For the assembly of lateral flow strips, we used NC140 nitrocellulose membrane (Sartorius, Goettingen, Germany) and AP 045 absorbent pads (Advanced Microdevices Pvt. Ltd., Ambala Cantt, India). In this study, for quantitative LFIA, we used commercial (Estapor^®^) particles with a diameter of 200 nm consisting of a number of superparamagnetic iron oxide nanocrystals entrapped in a polystyrene matrix, the surface of which is modified with COOH (carboxyl) groups. The working buffer solution was prepared using PBS, 1% BSA (Dia-m, Moscow, Russia), 0.1% Triton X-100 (Sigma Aldrich, St. Louis, MO, USA), and 10% casein (SDT GmbH, Baesweiler, Germany). Reagents A and B were sourced from Thermo Fisher Scientific, Waltham, MA, USA for the BCA protein assay. Monoclonal antibodies against folic acid (clone FA-1) were obtained from the Research Center of Molecular Diagnostics and Therapy, Russia, for adsorption competitive studies.

### 4.2. Spectral-Phase Interferometry Analysis

The kinetic properties of the antibodies were assessed using the spectral-phase and spectral-correlation interferometry methods [49,50,51,57]. The experimental setup consists of an optical system based on low-coherence interferometry, a fluidic system, and digital equipment necessary for signal registration and analysis. This setup enables the real-time measurement of changes in the optical thickness of the biolayer on the surface of the sensor chip, which result from molecular interactions.

Microscope cover glasses were chemically modified to create a sensor chip for kinetic studies. Initially, amino groups were introduced to the surface [58]. For this purpose, prewashed glasses were incubated overnight in a 1% APTES solution in a mixture of ethanol and water (95:5, *v*/*v*). After that, the glasses were washed 3 times with DMSO. Subsequently, the glass surface was modified using a succinic anhydride solution (60 mg in 40 mL of DMSO) to introduce carboxylic groups (the incubation time was 2 h). After carboxylation, the glasses were washed with DMSO 3 times and then dried in a dry-air sterilizer at 50 °C.

The BSA-ZEA conjugate was covalently bound to the carboxylic glass surface using the 3-step carbodiimide method. In the first step, 900 μL of 1% EDC-MES solution was applied to the glass surface for 1 h. After incubation, the glass was washed 3 times with Milli-Q grade water. The second step was to apply 900 μL of a solution of BSA-ZEA in PBS at a concentration of 20 µg/mL (the incubation time was 1 h). Then, the glass was washed 3 times with water again. The final step was to cover the glass with a 0.1 M Tris-HCl solution to neutralize the excess of activated carboxylic groups (the incubation time was 40 min). Finally, the glass was washed 3 times with water again. The chip was then integrated into the fluidic system of the label-free device, enabling the registration of changes in biolayer thickness resulting from intermolecular interactions between the immobilized conjugate and monoclonal antibodies in the solution.

The next step involved introducing a 150 µL sample of anti-ZEA antibodies in PBS at a concentration of 20 µg/mL. These antibodies were passed through the fluidic system, resulting in the registration of a sensorgram arising from the interaction between the antibodies and the immobilized conjugate, as well as a subsequent drop caused by the dissociation of the immune complex. Using the same procedure, we evaluated the specificity of the anti-ZEA antibodies. To achieve this, sensor chips were functionalized with various conjugates of carrier proteins and small molecules.

### 4.3. Functionalization of Magnetic Nanoparticles

Magnetic nanolabels were functionalized via two different methods. 

In the first approach, particles were functionalized by the carbodiimide method. Initially, 0.5 mg of COOH particles were activated using the EDC crosslinker in a 0.1 M MES buffer solution. Then, particles were washed 3 times with Milli-Q grade water by using the magnetic separation procedure. After that, a solution of anti-ZEA antibodies in PBS was added to the tube, and the mixture was incubated for 2 h. Following incubation, 5 µL of a blocking buffer (10% BSA in PBS) was introduced to ensure the colloidal stability of the particles and to prevent nonspecific interactions with proteins. Following a 2 h incubation with the blocking buffer, the particles underwent another round of magnetic separation, followed by resuspension in 100 µL of water. These functionalized nanolabels were stored at +4 °C until their intended use.

In the second approach, antibodies in PBS (pH 7.4) were added to 0.5 mg of COOH particles and incubated overnight for non-covalent binding. The resulting functionalized non-covalent labels were also stored at +4 °C before use.

### 4.4. BCA Assay

A BCA assay was performed to measure the part of antibodies which were adsorbed on the nanoparticles as a result of interaction in the solution. The analysis was performed in accordance with the manufacturer’s instructions [59]. In brief, 25 µL of the supernatant remaining after the particle functionalization procedure was combined with a mixture comprising 200 µL of reagent A and 4 µL of reagent B. These samples were then incubated for 30 min at 37 °C. After cooling the plate to room temperature, the absorbance at 562 nm was measured using an FM-96 spectrophotometer Berill (Biosan, Riga, Latvia).

### 4.5. Lateral Flow Assay Test Strip Fabrication

For the fabrication of LFIA strips, a nitrocellulose membrane measuring 300 × 20 mm was affixed to a PVC backing card. Subsequently, an absorbent pad was attached above the membrane, with a 2 mm overlap. The test line of the BSA-ZEA conjugate was dispensed onto the membrane using a density of 1 µL/min and a speed of 1 cm/min. After dispensing, the nitrocellulose card was dried at 37 °C for 1 h. Finally, the card was cut into strips measuring 2.5 mm in width using a paper guillotine. The obtained strips were stored at room temperature before use.

### 4.6. Magnetometric System and the Procedure of Analysis

The quantitative detection of magnetic nanolabels in our study was achieved through the implementation of the MPQ sensor. Previous studies have demonstrated the MPQ sensor’s ability to detect superparamagnetic materials with exceptional sensitivity, reaching sub-nanogram levels of magnetic material and an extraordinarily wide linear detection range spanning over seven orders of magnitude [41,42,43,60]. The detection mechanism is based on the nonlinear magnetization of nanoparticles in magnetic fields at two frequencies. The response, proportional to the amount of nonlinear magnetic material, is registered at combinatorial frequencies, which are algebraic sums with integer coefficients of the two excitation frequencies. Importantly, at these combinatorial frequencies, there is no contribution from linear para- or diamagnetic materials. This enables the achievement of high sensitivity and signal-to-noise ratio when measuring the quantity of magnetic nanoparticles.

For the development of the magnetic LFIA, strips containing printed BSA-ZEA test lines were employed. These strips were immersed in tubes containing 47 µL of the analyzed sample and 3 µL of magnetic labels. Following an incubation period of 20 min, the strips were scanned using the MPQ sensor to evaluate the distribution of magnetic nanolabels within the membrane volume. All experiments were conducted in three independent repetitions (*n* = 3), with the graphs showing the mean value; error bars represent the standard deviation (±SD).

### 4.7. Extraction of Zearalenone from Real Samples

For the extraction of zearalenone, 8 mL of a solution comprising acetonitrile and water (in a 60:40, *v*/*v* ratio) was added to 2 g of wheat. The mixture was incubated for 60 min. Subsequently, the supernatant was separated from the solid matrix via centrifugation (at 1500× *g* for 10 min) and the supernatant was further diluted at a 9:1 ratio.

## Figures and Tables

**Table 1:** Kinetic and equilibrium constants of the interaction of anti-ZEA Ab antibodies with BSA-ZEA conjugate and with free ZEA.

Target	k_on_, M^−1^s^−1^	k_off_, M^−1^s^−1^	K_d_, M	K_a_, M^−1^
BSA-ZEA	2.18 × 10^4^	5.08 × 10^−4^	2.33 × 10^−8^	4.29 × 10^7^
free ZEA	3.42 × 10^5^	7.05 × 10^−4^	2.06 × 10^−9^	4.85 × 10^8^

**Table 2:** Comparison of achieved analytical characteristics of the developed assay systems with alternative LFIA-based approaches.

Method	Assay Time, min	LOD, pg/mL	Dynamic Range, Orders	Ref.
MPQ-based LFIA, covalent conjugation	25	2.3	5.5	this study
MPQ-based LFIA, non-covalent sorption	25	7.6	5	this study
ICA with indirect labeling	17	5	3	[28]
ICA with MNPs	15	50	1	[38]
QB-ICA	10	62.5	2	[34]
MNPs-ICA	23	50	1	[39]
QB-ICA	15	59	2	[33]
ICA with Au@PDAs	30	7.4	2	[27]
ICA with PBNPs	6	100	1	[40]
Photothermal LFIA	13	4.3	5	[37]
FM-ICTS	15	480	1	[56]
Fluorescence quenchometric LFIA	20	100	1	[29]

**Table 3:** Detection of zearalenone in real barley flour samples using the developed magnetic LFIA.

Contaminated/ Uncontaminated Flour Ratio	ZEA (Expected), ng/mL	ZEA (Obtained), ng/mL	Recovery, %
100/0	35	32.9	94.0
75/25	26.25	27.5	104.8
50/50	17.5	16.3	93.1
25/75	8.75	8.6	98.3
0/100	0	not detected	n/a

## Data Availability

Data are contained within the article and Supplementary Materials.

## Supplementary Materials

The following supporting information can be downloaded at: https://www.mdpi.com/article/10.3390/toxins16010005/s1, Figure S1: DLS characterization of unconjugated (left), covalently conjugated (center), and non-covalently conjugated (right) particles; Figure S2: SEM images of the magnetic particles used in the study.
