# Supplementary material for: Quantitative Rapid Magnetic Immunoassay for Sensitive Toxin Detection in Food: Non-Covalent Functionalization of Nanolabels vs. Covalent Immobilization
Source: Toxins (Basel). 2023 Dec 20;16(1):5. doi: 10.3390/toxins16010005 (PMC10820704; doi:10.3390/toxins16010005)
Supplement: Supplementary file 1 [file toxins-16-00005-s001.zip › toxins-2719834-supplementary.pdf]

Supplementary Materials:

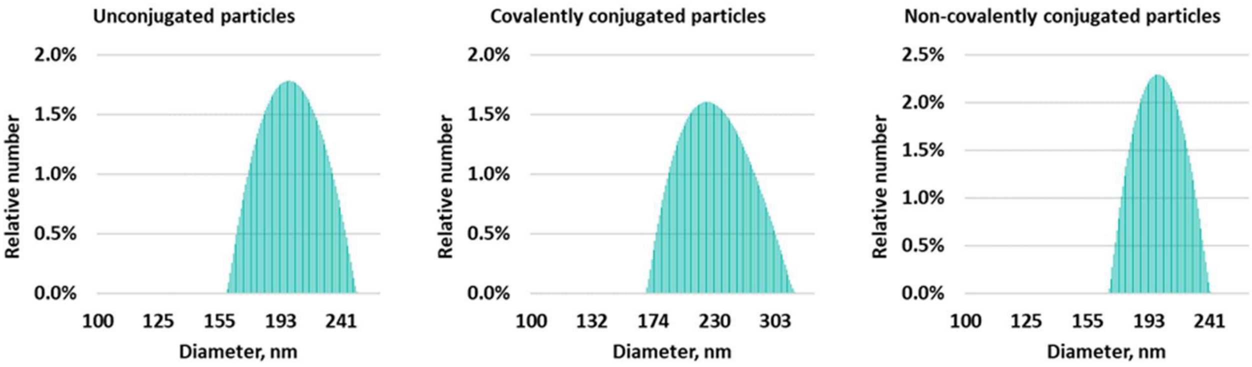

Figure S1. DLS characterization of unconjugated (left), covalently conjugated (center), and non-covalently conjugated (right) particles.

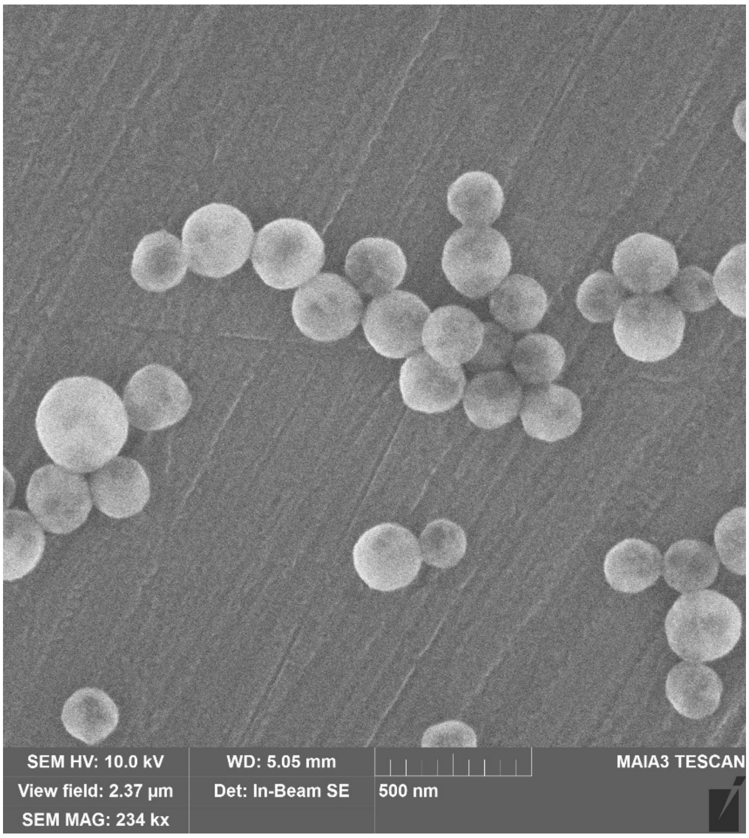

Figure S2. SEM images of the magnetic particles used in the study.
